# An Integrated Structural Model of the Palladin-Actin Complex Using XL-MS, SAXS, NMR, and Docking Approaches

**DOI:** 10.1063/4.0000842

**Published:** 2025-10-27

**Authors:** Moriah R Beck, Rachel A Sargent, David H Liu, Rahul Yadav

**Affiliations:** 1 Wichita State University, Wichita, KS, USA; 2 University of Arkansas-Ft. Smith, Fort Smith, AR, USA

## Abstract

Palladin is an actin-binding protein that accelerates actin polymerization and is implicated in the metastasis of multiple cancer types. Although three key lysine residues in an immunoglobulin-like domain of palladin have been identified as essential for actin binding, the precise binding site of palladin on F-actin remains unclear.

Our previous work suggests that palladin interacts with the sides of actin filaments to facilitate branching, as evidenced by its ability to compensate for Arp2/3 in *Listeria* actin comet tail formation. To elucidate the molecular basis of this interaction, we employed an integrative structural approach combining chemical crosslinking mass spectrometry (XL-MS), small-angle X-ray scattering (SAXS), NMR spectroscopy, and computational modeling. Crosslinking experiments covalently linked palladin and F-actin residues in proximity, followed by enzymatic digestion, liquid chromatography separation, and tandem mass spectrometry to identify crosslinked peptides. These constraints were then used as input for HADDOCK docking to model the palladin-F-actin complex. SAXS was employed to further refine the structural model, providing insights into palladin’s conformational flexibility and its binding interface with F-actin. Additionally, NMR spectra revealed potential domain-domain interactions within palladin that may contribute to its actin regulatory functions. Our final structural model of the palladin-F-actin complex demonstrates how palladin stabilizes actin filaments by interacting at the interface between two actin monomers. Notably, three actin residues identified in this study also appear in binding interfaces with other actin-associated proteins such as myotilin, myosin, and tropomodulin. By integrating SAXS with complementary structural techniques, we have developed the first comprehensive model of the palladin-actin complex. Given palladin’s role in promoting cancer metastasis through actin regulation, this structural insight may aid in the design of therapeutic strategies targeting this interaction to inhibit cancer cell motility and invasiveness.

